# Putative receptors and signaling pathways responsible for the biological actions of epoxyeicosatrienoic acids

**DOI:** 10.1016/j.jbc.2025.110737

**Published:** 2025-09-18

**Authors:** Matthew L. Edin, Joan P. Graves, Darryl C. Zeldin

**Affiliations:** Division of Intramural Research, National Institute of Environmental Health Sciences, National Institutes of Health, Research Triangle Park, North Carolina, USA

**Keywords:** Cytochrome P450, GPCR, epoxygenase pathway, EETs, epoxyeicosatrienoic acids, eicosanoid, receptor, signaling transduction

## Abstract

Epoxy fatty acids (EpFAs), including arachidonic acid (AA)-derived epoxyeicosatrienoic acids (EETs), are endogenously produced bioactive signaling molecules with diverse physiological effects, including vasodilation, anti-inflammation, and cardioprotection. EETs are generated by a subset of cytochromes P450 and their biological activity is reduced by hydrolysis to dihydroxyeicosatrienoic acids (DHETs) by epoxide hydrolases. Inhibition of soluble epoxide hydrolase (sEH) has shown significant therapeutic promise in preclinical models of disease. Despite the profound physiological impact of EETs and the therapeutic potential of sEH inhibitors, the precise signaling mechanisms by which EETs elicit their biological effects remain unknown. Many have sought to identify a high-affinity, EET-activated, G-protein-coupled receptor (GPCR). This review synthesizes current knowledge regarding the evidence supporting the existence of one or more EET-GPCRs and weighs this evidence against alternative or complementary EET signaling pathways. The breadth of these studies highlights the complexities and challenges in fully elucidating the precise molecular mechanisms of EET actions.

A subset of cytochromes P450 (P450s) can oxidize polyunsaturated fatty acids (PUFAs) to epoxy fatty acids (EpFAs) that are important endogenous signaling molecules. For example, some P450s metabolize arachidonic acid (AA) to epoxyeicosatrienoic acids (EETs) (1). EETs regulate a wide array of biological effects; they are vasodilatory, pro-angiogenic, anti-inflammatory, anti-apoptotic, anti-nociceptive, mitogenic, and they protect against ischemia-reperfusion injury (2). P450s can oxidize arachidonic acid on any of the four olefins to produce 14,15-, 11,12-, 8,9-, or 5,6-EET. There is no consensus as to which EET regioisomers are the most important *in vivo*. 14,15-EET is generally the most abundant measurable EET (3, 4); however, 11,12-EET may have the highest specific activity (5, 6). The effects of EETs are typically diminished by hydrolysis to less biologically active diols called dihydroxyeicosatrienoic acids (DHETs). Both soluble epoxide hydrolase (sEH) and microsomal epoxide hydrolase (mEH) hydrolyze EETs to DHETs *in vivo* (3). The potent physiological effects of endogenously produced EETs can be potentiated through the inhibition or genetic disruption of epoxide hydrolases (2, 3). GPCR screening for EET receptors has most commonly used 11,12- or 14,15-EET ligands or mimetics. These regioisomers are most increased by genetic disruption or pharmacological inhibition of sEH and are presumed to be the EETs responsible for altered physiology in most animal models (3). EETs are generated as both R,S and S,R stereoisomers (7); however, the data are conflicting as to whether either stereoisomer is more responsible for activation of a putative GPCR. For example, 14(S),15(R)-EET is more potent in vasodilation of blood vessels (8), while 14(R),15(S)-EET is more potent in membrane binding and cAMP formation in mononuclear cells (9).

It is worth noting the enormous potential of sEH inhibitors (sEHi) or EET mimetics for treating human diseases. Safe, effective sEHi or EET mimetic compounds have proven to be beneficial in a wide variety of preclinical models. For example, sEHi reduces the development of atherosclerosis and attenuates hypertension (10, 11). They improve cardiac recovery after acute ischemia (12), cardiac remodeling after myocardial infarction, reduce cerebral damage after stroke, and attenuate neurodegeneration and age-dependent cognitive decline (13). Pro-angiogenic effects of sEH disruption improve wound healing and enhance tissue regeneration (14). sEHi reduces inflammation and maintains blood pressure in models of septic shock (15). Their anti-inflammatory, anti-fibrotic, and anti-apoptotic effects attenuate organ damage from chronic conditions such as end-stage renal disease (16) and non-alcoholic fatty liver disease (17). They attenuate diabetes by reducing pancreatic beta cell apoptosis, improving insulin sensitivity, and preventing peripheral organ damage (18). sEHi demonstrated substantial benefit for stroke victims in a small clinical study and is currently being tested as a non-addictive treatment for neuropathic pain (19, 20). In short, they attenuate both acute stress and many diseases affected by chronic inflammation.

A major limitation in the EET research field and in the development of clinical applications for sEHi and EET mimetics is that we do not fully understand how EETs signal to induce their physiological responses. EETs are known to bind and activate the nuclear transcription factor peroxisome proliferator-activated receptors (PPARα and PPARγ) (21) and they can directly bind and/or activate ion channels, such as ATP-sensitive potassium channels (K_ATP_) or transient receptor potential (TRP) channels type C and type V (22, 23). A large body of evidence suggests that EETs elicit signals through one or more heterotrimeric G-protein-coupled receptors (GPCRs); however, to date, no high-affinity receptors have been discovered. Identification of a receptor or family of receptors could lead to the development of selective agonists or antagonists that may have clinical application to treat various diseases. In this review we will discuss the evidence that suggests the existence of a high-affinity EET-GPCR (EETR), the many attempts to identify this GPCR, and alternatives to the EETR signaling hypothesis.

## Evidence for an EETR

The concentration of individual, non-esterified EETs in plasma is low (∼1–10 nM) (24, 25, 26), while concentrations in tissues are 10-fold higher. EETs trigger physiological effects, including vasodilation, anti-inflammation, bronchodilation, and angiogenesis, at concentrations as low as 1 to 100 nM in a combination of *in vivo*, *ex vivo,* and *in vitro* assays, nicely summarized elsewhere (27). While the local concentrations that may induce EET effects are unknown, efforts have focused on identifying a high-affinity receptor (<100 nM) from the family of known, non-olfactory GPCRs.

Lipid signaling molecules can signal through GPCRs. High-affinity GPCRs have been reported for prostaglandins, P450- and lipoxygenase-derived hydroxyeicosatrienoic acids (HETEs), lipoxins, leukotrienes, and specialized pro-resolving mediators (28, 29, 30); however, the International Union on Basic and Clinical Pharmacology (IUPHAR) has strict standards for recognizing ligand/GPCR pairs. The IUPHAR requires identification of specific high-affinity binding, understanding of receptor physiology and pharmacology, and independent replication of results to rename orphan GPCRs and formally acknowledge ligand/GPCR pairs (31). To date, the IUPHAR has only officially recognized the high-affinity interactions of multiple prostaglandins and leukotrienes to cognate receptors, and the interaction of 12-HETE with GPR31 (32). Despite the many suggestive studies listed below, the IUPHAR has yet to formally recognize a receptor for most HETEs or any SPM or EpFA (32).

The binding of ligand to GPCRs induces GTP-loading, activation, and dissociation of Gα subunits from the receptor (33). Activation of a single receptor can lead to a variety of outcomes by coupling to different α subunits in different cell types. Four Gα subtypes induce a variety of cellular responses; Gα_s_ subunits stimulate adenylyl cyclase (AC) to induce cAMP formation and PKA activation, Gα_i_ subunits inhibit AC and induce cGMP phosphodiesterase, Gα_q/11_ subunits activate phospholipase C and increase intracellular calcium, and Gα_12/13_ subunits can activate Rho family GTPases (33). Gβγ subunits also activate signaling pathways such as the Extracellular Regulated Kinase (ERK) pathway (34). Given the structural similarity of EETs to other known GPCR ligands and their ability to stimulate many GPCR pathways *in vitro*, it seems both logical and likely that EETs may act as ligands to transactivate one or more GPCRs to induce downstream signals.

**Figure 1:**
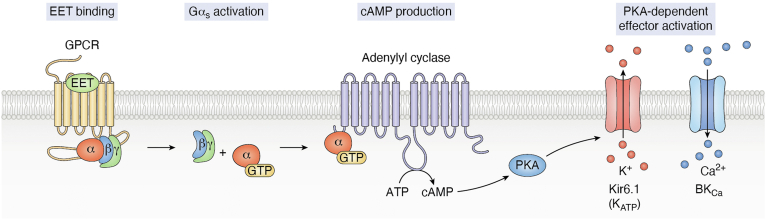
**Evidence for EET signaling through a G-protein coupled receptor.** EETs are known to bind a membrane receptor with high affinity, which results in canonical GTP loading of Gα subunits, activation of adenylyl cyclase to form cAMP and activate PKA-dependent regulation of ion channel opening.

EETs have been shown to elicit specific effects which suggest that they activate GPCR signaling (Fig. 1). EETs exhibit high-affinity membrane binding, induce Gα_s_ GTP-loading, transactivate cAMP induction, and regulate secondary effects *via* PKA. In 1997, Wong and co-workers first identified a high-affinity binding site for 14,15-EET in monocyte membranes. Radiolabled 14,15-EET studies revealed a half-maximal binding concentration (Kd) of 14 nM in U937 cells and of 35 nM in primary pig monocytes (9, 35). EETs activated cAMP in monocytes; however, this effect required supraphysiologic (micromolar) levels of 14,15-EET (9, 35). Evidence for an extracellular binding site for EETs was demonstrated using silica beads coated with cross-linked 14,15-EET. Despite the inability to cross the cell membrane, silica-linked 14,15-EET beads suppressed dibutyryl cAMP-induced aromatase induction to a similar degree as free 11,12- or 14,15-EET (36). EETs require ADP-ribosylation of Gα_s_ to activate vascular K_ATP_ channels (37). Li *et al.* also determined that EETs regulate smooth muscle relaxation *via* Gα_s_ signaling. They demonstrated that 11,12-EET-induced activation of large-conductance Ca^2+^-activated potassium channel (BK_Ca_) required GTP-loading of Gα_s_ and activation of PKA. This signaling was induced by low nanomolar concentrations of 11,12-EET (38). All of these data suggest binding and activation of an EET-responsive GPCR.

## EETR screening efforts

Multiple labs have tried different methods to identify the putative EETR. Methods to reveal direct EETR binding involve incubation of membranes or cells with labeled EETs, including biotinylated, photoaffinity cross-linkable or radiolabeled EETs (18, 39). An alternative method to detect direct receptor interaction involves displacement of a known ligand by addition of EETs, or displacement of EETs by a known ligand (40). Indirect methods to identify the EETR include transfection of cloned GPCRs or incubation with GPCR inhibitors coupled to the detection of cAMP formation, calcium influx, ERK activation, ion currents, β-arrestin translocation or arterial vasodilation (41, 42). While indirect methods may have advantages in high-throughput screening assays, they must be confirmed with direct binding assays to determine the affinity of EETs as ligands to putative GPCRs.

Several examples of unsuccessful EETR cloning efforts have been published. Given the bias against the publication of negative results, it is likely that other unsuccessful cloning attempts have not been reported. In 2007, the Hammock lab contracted with the CEREP Laboratories (https://www.eurofins.com/) to evaluate whether EETs could displace known ligand/receptor interactions in a series of 43 GPCRs involved in pain sensation (Table 1). Of these, EETs were found to displace ligands from the peripheral benzodiazepine receptor, cannabinoid CB_2_ receptor, neurokinin NK_2_ receptor, and dopamine D_3_ receptor; however, displacement required >10 μM EETs. The authors concluded that none of these receptors was likely a high-affinity EETR (40). In 2011, Chen *et al.* reported the use of a radiolabeled photoaffinity EET compound that could be activated by UV light to crosslink to adjacent proteins. The authors detected crosslinking of this compound to a 47 KDa protein in smooth muscle cells and endothelial cells from rabbit, human, and bovine arteries. Crosslinking was prevented by the addition of 8,9-, 11,12-, or 14,15-EET (39). 47 kDa is a common size for a GPCR; however, the authors were unable to identify the labeled protein. They pursued this finding by transfecting 80 orphan receptors into HEK293 cells; however, they were unable to reproduce specific crosslinking to any of these receptors (Table 2) (39). Our laboratory contracted with the Roth laboratory at the University of North Carolina, which can screen for activation of GPCRs using PRESTO-Tango technology (43). This screen failed to detect activation of any of 347 GPCRs using 1 μM concentrations of 8,9-, 11,12-, and 14,15-EET; however, these methods may not be ideal for the identification of oxylipin receptors, as they also failed to identify GPR75 as a 20-HETE receptor.

**Table 1:** GPCRs examined by CEREP for the ability of EETs to displace known radioligands from human receptors that may be involved in pain sensation (Inceoglu *et al.*, POLM, 2007)

Receptors			
5-HT	CB1	Glycine (strychnine-insensitive)	NK2
A1	CB2^a^	Glycine (strychnine-sensitive)	NK3^a^
alpha 1	CCR1	H1	NMDA
alpha 2	CCR2	H2	NT
AMPA	CCR3	I1	Opioid
ANP	CGRP	I2	ORL1
AT1	CXCR2	Kainate	P2X
AT2	CXCR4	M	P2Y
B1	D1	N (muscle-type)	Sigma
B2	D2S	N (neuronal) (BGTX-insensitive)	Sst
beta 1	D3^a^	N (neuronal) (BGTX-sensitive)	Y
BZD (peripheral)^a^	GABA	NK1	

a Receptors that showed low affinity (10–100 μM) displacement of endogenous receptor radioligands.

**Table 2:** GPCRs transfected into HEK293 cells and examined for crosslinking by a photoactivatible EET (Chen *et al.*, Biochemistry, 2011)

Receptors				
ADMR	GPR26	GPR75	GPR133	GPR176
CD97	GPR31	GPR78	GPR135	GPR182
EB12	GPR32	GPR80	GPR139	GRCA
EMR1	GPR34	GPR82	GPR143	MAS1
ETLD1	GPR35	GPR83	GPR146	MAS1L
G2A	GPR37L1	GPR84	GPR148	MRGD
GPR1	GPR40	GPR85	GPR151	MRGE
GPR3	GPR41	GPR87	GPR153	MRGF
GPR6	GPR43	GPR88	GPR155	MRGX3
GPR12	GPR45	GPR89	GPR160	MRGX4
GPR15	GPR52	GPR97	GPR161	OPN3
GPR18	GPR56	GPR101	GPR171	P2RY8
GPR19	GPR61	GPR107	GPR172 A	P2Y10
GPR21	GPR62	GPR114	GPR173	PSGR
GPR22	GPR63	GPR116	GPR174	RAIG3
GPR25	GPR64	GPR120	GPR175	TM7SF1

No GPCRs were identified as positive for EET binding.

Several aspects of GPCR physiology make it challenging to identify putative EET receptors. For example, some agonists only activate GPCRs that exist as heterodimers (44). Identifying an EET-sensitive GPCR that acts only as a minimal functional unit of a heterodimer would require the happenstance of screening singly-transfected cells that express sufficient levels of the heterodimer partner. Similarly, some GPCRs interact with Receptor Activity-Modifying Proteins (RAMPs) that interact with GPCRs to modify their function. Binding with different RAMPs can regulate the sensitivity of GPCRs to ligand to alter signaling and/or trafficking in a receptor-dependent manner (45). Furthermore, attempts to deduce an EETR by RNA-expression profiles of EET-sensitive cell types may be confounded by the existence of a family of receptors capable of responding to EETs; no single gene would necessarily be expressed in every EET-sensitive cell line or cell type. EETs may also regulate GPCRs indirectly, like the recent finding that resolvins have anti-inflammatory activity through allosteric modification of PGE_2_ activity on the EP4 receptor (46). Lastly, while it is presumed that free, non-esterified EETs are the putative signaling molecule, alternative epoxide metabolites, such as epoxyendocannabanoids are regulated by similar P450 and epoxide hydrolase pathways and may have independent or overlapping effects (47).

## Identified EETR activities

### GPR40/GPR120 agonist

Many studies have identified EpFA binding or activities through GPCR-dependent mechanisms (Table 3). The first mention of an EET transactivating a known GPCR came in 2003 when Itoh and coworkers found that free fatty acids regulate insulin secretion through GPR40. While it is unclear how many fatty acids and oxylipins they screened, they reported that 11,12-EET and 14,15-DHET increased intracellular calcium levels in GPR40-transfected CHO cells with EC50s of 1.4 μM and 1.1 μM, respectively. 5,6- and 8,9-EET were less potent, increasing calcium levels with EC50s of 7.7 μM and 6.1 μM, respectively. 11,12-EET was equipotent as docosahexaenoic acid (DHA), while AA activated GPR40 with an EC50 of 2.4 μM. A family of GPCRs are known to be activated by a variety of free fatty acids and are thus termed free fatty acid receptors (FFARs). GPR40 is also known as FFAR1 (48).

**Table 3:** Activities of oxylipins identified against selective GPCRs

GPCR	Ligand(s)	Assay	Activity	Reference
GPR40	14,15-DHET	Intracellular Ca	1.1 μM	Itoh *et al.*, 2003
	5,6-EET		1.4 μM	
	8,9-EET		6.1 μM	
	11,12-EET		7.7 μM	
	AA		2.4 μM	
	11,12-EET	ERK activation	5 μM	Ma *et al.*, 2015
	14,15-EET		5 μM	
	5,6-EET	Intracellular Ca	∼3 μM	Park *et al.*, 2015
	8,9-EET		∼3 μM	
	11,12-EET		0.91 μM	
	14,15-EET		0.58 μM	
	11,12-DHET		>10 μM	
	14,15-DHET		>10 μM	
	17,18-EpETE	TGFα shedding	10 μM	Aoki *et al.*, 2023
	19,20-EpDPA		0.3 μM	
GPR120	8,9-EET	Intracellular Ca	>5 μM	Park *et al.*, 2015
	11,12-EET		>5 μM	
	14,15-EET		>5 μM	
	17,18-EpETE	TGFα shedding	10 μM	Aoki *et al.*, 2023
	19,20-EpDPA		0.3 μM	
GPR132	11,12-EET	Arrestin recruitment	∼2 μM	Lahvic *et al.*, 2018
	14,15-EET		∼10 μM	
GPR39	14,15-EET	ERK activation	100 nM	Alkayed *et al.*, 2022
	5,6-EET		None	
	8,9-EET		None	
	11,12-EET		None	
TP	5,6-EET	Vasorelaxation	1.7 μM	Behm *et al.*, 2009
	8,9-EET		0.6 μM	
	11,12-EET		0.6 μM	
	14,15-EET		0.8 μM	
	5,6-DHET		1.0 μM	
	8,9-DHET		1.2 μM	
	11,12-DHET		0.5 μM	
	14,15-DHET		1.8 μM	
	11,12-EET	Vasorelaxation	0.3 μM	Malacarne *et al.*, 2022
	14,15-EET		0.3 μM	
	8,9-EET	Bronchorelaxation	1 μM	Senouvo *et al.*, 2011
	14,15-EET		0.1–1 μM	
EP2	14,15-EET	Radioligand binding	>10 μM	Behm *et al.*, 2009
	14,15-EET	Oocyte conductance	1 μM	Liu *et al.*, 2016
	14,15-EET	ERK activation	1 μM	
	14,15-EET	Vasorelaxation	10 μM	Yang *et al.*, 2010
	14,15-EET	Vasorelaxation	3 μM	Yang *et al.*, 2014
	11,12-EET	cAMP formation	1 μM	Matsumoto *et al.*, 2022
	14,15-EET		1 μM	
	8,9-DHET		1 μM	
	9,10-EpOME		1 μM	
	12,13-EpOME		1 μM	
EP3	14,15-EET	Oocyte conductance	1 μM	Liu *et al.*, 2016
EP4	14,15-EET	Oocyte conductance	0.1 μM	Liu *et al.*, 2016
	14,15-EET	ERK activation	1 μM	
	14,15-EET	ERK activation	1 μM	Nagarajan *et al.*, 2021
S1PR1	17,18-EpETE	Intracellular Ca	0.1 μM	Zhou *et al.*, 2024

Oxylipins examined for activities in cells or tissues is listed by tested GPCR. Concentration listed is IC50 or EC50 if determined by the authors or concentrations where statistically significant effects were observed. See the review text for details regarding assays performed.

Subsequently, Ma *et al.* demonstrated that both 11,12- and 14,15-EET could transactivate the ERK pathway in GPR40-transfected cells. In their system, GPR40 was suggested to activate a sheddase that released heparin-bound epidermal growth factor-like growth factor (HB-EGF) to activate the EGF receptor (EGFR). These studies required relatively high levels of EETs, as no ERK phosphorylation was observed at less than 5 μM of either EET (49).

Park *et al.* also demonstrated EET signaling in HEK cells transfected with GPR40. Both 11,12- and 14,15-EET increased intracellular calcium levels at doses less than 1 μM (0.91 μM and 0.58 μM, respectively). Signaling by 11,12- and 14,15-EET was noticeably stronger than 5,6- or 8,9-EET, 11,12- or 14,15-DHET and the eicosapentaenoic acid (EPA) epoxide, 17,18-EpETE. Importantly, these authors demonstrated direct binding to the receptor by showing displacement of the known GPR40 agonist TAK-875. 8,9-, 11,12- and 14,15-EET also increased calcium levels in HEK cells transfected with GPR120 (FFAR4); however, GPR120 activation required significantly higher levels of these EETs (5–10 μM). While GPR40 appears to be most abundantly expressed in the pancreas (50), this study demonstrated both GPR40 and GPR120 expression in both endothelial and smooth muscle cells (51).

In summary, multiple independent groups have demonstrated that EETs have GPR40 agonist activity. GPR40 is expressed in EET-sensitive cell types, such as endothelial, smooth muscle and pancreatic cells. EETs were shown to directly bind to GPR40; however, the estimated 500 nM-5 μM range of Kds suggests that neither GPR40, nor GPR120 are high-affinity EET receptors. Given that most stimuli that trigger release and/or formation of EETs will also release much larger quantities of many free fatty acids, it remains unclear to what extent EETs contribute to GPR40 or GPR120 signaling *in vivo*. Nonetheless, GPR40 signaling parallels EET signaling in many vascular phenotypes. GPR40 signaling induces angiogenesis (52), is anti-nociceptive (53), reduces inflammation *via* inhibition of NF-κB (54), prevents neurodegeneration (55), and protects hearts against ischemia-reperfusion injury (56). Since GPR40 signaling can induce selective, ligand-dependent activation of alternative pathways (54), discerning the role of EET signaling through GPR40 receptors *in vivo* is likely to be complex.

### GPR132 agonist

Lahvic *et al.* screened for EET-sensitive GPCRs after finding that 11,12-EET promoted hematopoietic stem cell engraftment in a zebrafish transplant model (57). Screening GPCR-transfected cells using a hybrid β-arrestin recruitment system, they found that 11,12-EET activated GPR132 which resulted in its phosphorylation and β-arrestin recruitment. GPR132 was found to be activated by both 11,12-EET and 11,12-DHET, though with relatively high EC50s of 2 to 10 μM. GPR132 was tested for responses to a variety of oxylipins and fatty acids and was found to be most strongly activated by 9-HODE, similar to previous studies (58). 11,12 EET activated GPR132 in this system to the same degree as AA. Given the relative abundance of 11,12-EET compared to 9-HODE and AA, these data would suggest that GPR132 is most likely not primarily responding to EETs *in vivo*. Since GPR132 is activated my multiple oxylipins and fatty acids, it is now recognized as a less well-characterized member of the FFAR superfamily (59).

### GPR39 agonist/antagonist

14,15-EET was recently identified to bind to GPR39 *via* a 14,15-EET photoaffinity probe similar to the one described above (39). Alkayed *et al.* demonstrated that 14,15-EET induced ERK phosphorylation only in GPR39-transfected HEK293 cells (18). The 14,15-EET-induced activation of ERK was potentiated by an order of magnitude by the addition of 1 μM zinc. Neither 11,12-, 8,9-, nor 5,6-EET showed any activity in GPR39-transfected cells. Docking simulations successfully identified that 15-HETE may also bind GPR39. In the presence of zinc, both 14,15-EET and 15-HETE transactivate ERK signaling *via* GPR39 with EC50s of less than 100 nM. *Ex vivo* coronary perfusion experiments in wild-type and GPR39 knockout hearts revealed complex interactions between 14,15-EET, 15-HETE, and GPR39. 1 μM 15-HETE induced coronary vasoconstriction and elevated coronary perfusion pressure in hearts from wild-type mice. 15-HETE-induced increases in coronary perfusion pressure were abolished in GPR39-deficient mice. 14,15-EET apparently had no effect on coronary artery tone by itself; however, 14,15-EET antagonized the coronary vasoconstriction induced by 15-HETE. Given that GPR39 had unusual selectivity and responses to 14,15-EET, the authors concluded that GPR39 is not the single, high-affinity, EET receptor; however, it may belong to a family of EET receptors through which EETs exert their physiological effects (18).

Experiments in GPR39-deficient mice suggest many parallels between GPR39 and EET signaling. While potentiation of EET signaling improves microvascular flow and cerebral outcomes in a murine stroke model, GPR39 null mice have worse brain injury, microvascular perfusion, and neurological function after experimental stroke (60). In contrast, GPR39-deficient mice have increased sensitivity to inflammatory colitis and impaired glucose tolerance, insulin secretion and wound healing (61). These effects in GPR39-deficient mice are similar to the effects seen by potentiation of EET signaling, which suggests that GPR39 may mediate some of the EET signaling that occurs *in vivo* (14, 62, 63).

### Thomboxane receptor antagonist

Several reports suggest that EETs may act as selective antagonists to thromboxane receptors (TP receptors). Two groups have reported that EETs relax arteries preconstricted with the TP agonist U46619. In 2009, Behm *et al.* found that EETs could relax rat aorta or mouse mesenteric arteries preconstricted with the TP receptor agonist U-46619, but not arteries preconstricted with phenylephrine, endothelin-1, or potassium chloride. 8,9- and 11,12-EET were the most potent oxylipins, inducing 50% relaxation (IC50) at concentrations of 0.6 μM. 14,15-EET was slightly less potent with an IC50 of 0.8 μM. 8,9- and 14,15-DHET demonstrated significantly less relaxation than their corresponding EETs; however, 11,12-EET and 11,12-DHET induced similar relaxation of U46619 preconstricted arteries (64). This pattern of oxylipin potencies almost exactly matches the potency of these oxylipins in relaxing human corornary arteries (5). 14,15-EET could also attenuate U46619-mediated constriction of rat tertiary bronchioles. Importantly, the authors demonstrated that 14,15-EET could displace specific binding of radiolabled SQ-29548 (a TP agonist) with an IC50 of 3.3 μM. 14,15-EET could also displace radioligands from PGF_2α_ receptors (FP, IC50 5.3 μM) and PGD_2_ receptors (DP, IC50 6.1 μM). 14,15-EET could not displace radioligands for prostacyclin, PGE_2_, or leukotriene receptors (IP, EP1-4, BLT2, CysLT1-2, respectively) at concentrations less than 10 μM. Malacarne *et al.* found similar findings examining mouse aortic responses; micromolar concentrations of 11,12-EET could relax aortas preconstricted with U46619, but not aortas preconstricted with phenylephrine (65). Senouvo *et al.* also found that U46619-precronstricted bronchioles could be relaxed by either 14,15- or 8,9-EET at concentrations of 0.1 to 1 μM or >1 μM, respectively (66).

The three independent laboratories report similar selective relaxation of TP-agonist preconstricted arteries or bronchioles, which implies that EETs act as selective TP receptor antagonists. While these findings are unequivocal, it is unclear the extent to which EETs act as TP antagonists *in vivo*. EETs or sEHi relax vessels and/or decrease blood pressure in some models not primarily reliant on thromboxane-induced vasoconstriction (5, 10). TP receptor antagonism is also intriguing as it could also partially explain the mechanisms underlying anti-inflammatory effects of EETs. TP receptors are broadly expressed throughout the cardiovascular system (67); however, the relatively low-affinity antagonism of thromboxane-TP-receptor interactions is unlikely to mediate many other EET-mediated effects, including their angiogenic, mitogenic, promigratory, anti-apoptotic, and cytoprotective properties (1).

### PGE_2_ receptor agonist

Multiple reports suggest the possibility that EETs may act through PGE_2_ receptors. The four established PGE_2_ receptors act through different pathways. EP2 and EP4 stimulate cAMP and PKA, while EP4 also transactivates PI3K and ERK signaling. EP1 activates PKC and increases intracellular calcium levels, and EP3 suppresses cAMP formation (68). As noted above, Behm *et al.* determined that EETs had low affinity (>10 μM) for all 4 EP receptors (64). In contrast, Liu *et al.* reported the results of a broad screen of GPCRs transfected into frog oocytes with a reporter signal based on detection of cystic fibrosis transmembrane conductance regulator (CFTR) transactivation (41). EP2, EP3, EP4, FP, and DP receptors all produced responses to 1 μM 14,15-EET, but only EP4 responded to as little as 100 nM 14,15-EET. In follow-up experiments, HEK293 cells expressing EP2 and EP4 significantly increased ERK phosphorylation after treatment with 1 μM 14,15-EET. In contrast, FP-, DP- and EP3-transfected cells failed to activate ERK in response to 14,15-EET. EET responses were not blocked by indomethacin, which suggests that EP2 and EP4 responses are direct interactions, not secondary to EET-stimulated formation of prostaglandins (41).

The potential role for EP receptors in modulating the effects of EETs is bolstered by additional studies. Yang *et al.* demonstrated that rat mesenteric arteries preconstructed with phenylephrine could be relaxed by 14,15-EET. While these relaxations required high EET levels (∼10 μM 14,15-EET), the EET-induced relaxation was significantly inhibited by the EP2 receptor antagonist AH6809, but not by EP1, EP3, EP4 antagonists. In addition, specific binding of 14,15-EET to rat smooth muscle cells was significantly reduced in cells pretreated with EP2-specific siRNA (69). In a separate study, the ability of 14,15-EET to induce relaxation of mesenteric arteries was reduced in arteries from aged mice. This finding is concurrent with a reduction in mesenteric arterial EP2 receptor expression as mice age. Regardless of age, EP2 antagonists were able to reduce the ability of 14,15-EET to relax preconstructed mesenteric arteries (70).

Matsumoto *et al.* investigated the induction of cAMP formation by a panel of oxylipins in several cell types (71). They found that 1 μM concentrations of 14,15- and 11,12-EET, 8,9-DHET, both EpOMEs and AA most consistently induced cAMP formation in HEK293, human coronary artery smooth muscle (HCASMC), and LLC-PK1 cells. In HEK293 and LLC-PK1 cells, EET-induced cAMP formation could be strongly reversed by the EP2-receptor antagonist TG6-10-1, while the EP4 receptor antagonist had little effect. In contrast, neither antagonist reversed EET-induced cAMP formation in HCASMC, which may lack EP2 and EP4 receptors. Interestingly, HCASMC induced cAMP formation by 8,9-DHET was attenuated by siRNA knockdown of the prostacycling receptor (IP). cAMP formation induced by AA could be strongly inhibited by the COX-2 inhibitor diclofenac, which suggests that AA was not itself a signaling ligand, but was converted to one or more prostaglandins that induced signal transduction. The EET and EpOME-induced formation of cAMP was also substantially reduced by dicolofenac; thus, it remains unclear whether EET transactivation of EP2 receptors was by direct interaction with a receptor or through the formation of PGE_2_ or other prostaglandins which act as second messengers (71).

Nagarajan *et al.* also identified a PGE_2_ receptor (EP4) in a panel of 111 GPCRs transfected into HEK 293 cells and screened for 1 μM 14,15-EET-induced ERK activation (42). EP4 was the strongest hit in this assay; however, it was only marginally better than other reported hits, which included CCR3, GPR85, GPR17, CXCR4, PAR1, GPR31, IP, and GPR63. The authors did not evaluate direct EET-receptor binding or interrogate whether EETs triggered release of known ligands for these receptors, which include chemokines such as eotaxin, RANTES, MCP-3/4, and CXCL12, and lipids such as 12(S)-HETE, PGI_2_, LTC_4_, and S1P. Nonetheless, these authors provide an intriguing list that should be independently validated for regulation of EET-induced *in vitro* signaling and *in vivo* physiology.

### Other EpFA-activated GPCRs

The most abundant EPA-derived EpFA, 17,18-epoxyeicosatetraenoid acid (EpETE), was recently identified as a ligand for the sphingosine-1-phosphate receptor-1 (S1PR1) (72). 17,18-EpETE displaced [^3^H]-S1P from S1PR1 and demonstrated potent effects *in vitro*, regulating calcium influx and endothelial activation at 10 to 100 nM. Importantly, the ability of 17,18-EpETE to attenuate the development of atherosclerosis *in vivo* was abolished in S1PR1-deficient mice. The authors ruled out involvement of GPR132 and GPR120 in 17,18-EpETE activity; however, they did not examine whether EETs activated S1PR1. Interestingly, EETs were previously demonstrated to activate sphingosine kinase, the enzyme that generates S1P (73). Thus, there is a possibility that a complicated synergy between EpETE and S1P occurs on S1PR1. While EET transactivation of S1PR1 remains possible, a previous study suggests that EET and EpETE signaling may be distinct; 14,15-Epoxyeicosa-5(Z)-enoic acid (EEZE), a putative EET receptor antagonist, fails to block vasodilation induced by 17,18-EpETE (74). 17,18-EpETE is also reported to transactivate GPR40 and GPR120 to regulate contact hypersensitivity; however, these studies indicate that these are lower affinity interactions with EC50s of >2 μM and >10 μM, respectively (75).

The most abundant DHA-derived EpFA, 19,20-epoxydocosapentaenoic acid (EpDPA), appears to act through the FFARs GPR40 and GPR120. Aoki *et al.* report that 19,20-EpDPA attenuates non-alcoholic fatty liver disease (NAFLD) and liver fibrosis, an effect that can be reversed by GPR120 antagonists. 19,20-EpDPA activated TGFα shedding in both GPR40-and GPR120-transfected cells, though maximal effects were in the concentration range (10–33 μM) at which moxt oxylipins assayed, including omega-3 diols, had some effects. 19,20-EpDPA most strongly activated GPR40 at a concentration of 330 nM, though only GPR120 inhibitors or *Gpr120* genetic disruption altered the ability of 19,20-EpDPA to attenuate NAFLD. More detailed receptor-binding kinetics and follow-up experiments are warranted (76).

### Effective low-affinity interactions

While other EET-like lipids are known to signal through high-affinity GPCRs, it remains possible that no high-affinity, EET-sensitive GPCR exists. *In vivo*, EETs may effectively transactivate GPCRs *via* lower-affinity interactions in situations of high local EET concentrations *via* coordinated release of EETs. Our understanding of how EETs are formed and transport to induce receptor signaling is incomplete; however, *in vivo* EET formation and processing may be significantly different from that of prostaglandins. Both the cyclooxygenases and synthases that generate prostaglandins and P450s that generate EETs are largely confined to the endoplasmic reticulum (77, 78). Once formed, prostaglandins or free EETs can be bound by fatty acid binding proteins and lipid transporters to protect them against degradation and/or facilitate extracellular efflux (79, 80). Interestingly, while most prostaglandins bind extracellular sites on GPCRs, the binding site of fatty acids to GPR40 was recently proposed to be on amino acids in the transmembrane sections that lie within the lipid bilayer and adjacent to the cytoplasmic face of the lipid bilayer (81). 14,15-EET binding to GPR39 was also modeled to occur at transmembrane sites (18). Like many oxylipins, most EETs in a cell are esterified to the *sn-2* position of membrane phospholipids (82). Esterified EETs may become distributed throughout cell membranes and can be preferentially released by the same stimuli and PLA_2_ enzymes that release AA (82, 83). It is unclear whether EET signaling occurs directly after EET formation or after PLA_2_-mediated release. The binding site may even be best accessed by AA released from the cytosolic side of the membrane. Thus, there remains the possibility that esterified EETs could be released from plasma membrane phospholipids to produce very high local concentrations adjacent to EET-sensitive GPCRs.

## Non-EET-selective GPCR signaling mechanisms

### Indirect receptor crosstalk

Transactivation of lipid receptors is not unusual. For example, the formation of cAMP in fibroblasts after stretch relaxation has been traced to activation of TRPV4 channels, increases in intracellular calcium, activation of PLA_2_, liberation of AA, and COX-dependent prostaglandin signaling (84, 85). While multiple investigators have proposed a role of prostaglandin receptors in EET signaling, the evidence is conflicting as to whether EETs may transactivate EP, DP, FP, or IP receptors directly or *via* COX-dependent formation of prostaglandins (41, 71).

In addition to the possibility that EETs transactivate prostaglandin signaling, there are reports of EETs transactivating other receptor pathways. Chen *et al.* first reported that 14,15-EET induced transactivation of EGFR to induce mitogenic ERK signaling (86). The initiating factor may be activation of a GPCR such as GPR40 (49). Using a series of inhibitors, 14,15-EET-induced activation of ERK was shown to require activation of metalloproteinase activity to release membrane-bound proHB-EGF, which activated the EGFR (86). EETs are mitogenic, angiogenic, anti-apoptotic, and cardioprotective (2), consistent with the known role of HB-EGF and ERK signaling. HB-EGF has wide-ranging effects throughout the body and regulates tissue repair and regeneration as well as tumor growth and metastases, similar to effects seen in high-EET mice (14, 87, 88, 89).

Terashvili *et al.* demonstrated that, while 14,15-EET fails to directly bind μ- or δ-opiod receptors, it activates β-endorphin or Met-enkephalin signaling that can act on these receptors to reduce pain (90). In a separate study, 11,12-EET induced cardioprotection indirectly through a mechanism that was blocked by inhibition of κ-opiod receptor signaling (91). Similarly, sEH null or 11,12-EET-treated hearts were found to increase levels of B-type natriuretic peptide (BNP) after cardiac ischemia-reperfusion injury. This release of BNP was thought to be significant, as the improved post-ischemic recovery of sEH null or 11,12-EET-treated hearts could be attenuated by the natriuretic peptide receptor-A receptor antagonist A71915 (92).

### GPCR-independent EET signaling pathways

**Figure 2:**
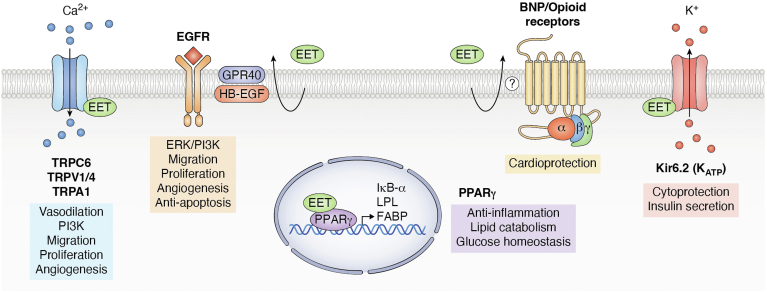
**Alternative EET-sensitive pathways.** EETs can bind and activate TRP channels that can induce both vasodilation and PI3K activation to regulate cell migration, proliferation, and angiogenesis. EETs can activate Kir6.2-containing K_ATP_ channels to induce potassium efflux that can regulate cardiac and neuronal cytoprotection as well as insulin secretion. EETs also bind the PPAR nuclear receptor which induces genes that inhibit inflammation or promote lipid catabolism and glucose homeostasis. EETs may also be involved in receptor transactivation. EET treatment results in activation of B-type natriuretic peptide (BNP) or opiod receptors by unknown mechanisms which results in cardioprotection. EETs can act through GPR40 to release heparin-bound Epidermal Growth Factor (HB-EGF) to transactivate EGF receptors, activate ERK and PI3K pathways, and which can regulate migration, proliferation, angiogenesis and apoptosis.

It is possible that some, many or all of the many effects of EETs may be orchestrated by known GPCR-independent signaling mechanisms. EETs are known to directly bind PPARs and regulate the opening of TRP and K_ATP_ channels. While not always studied in the context of EET treatment, such pathways are known to regulate numerous physiological effects canonically associated with EETs, including their anti-inflammatory, mitogenic, angiogenic, cardioprotective, and vasodilatory effects (Fig. 2).

Multiple studies demonstrate that EETs act as PPARα and PPARγ agonists in reporter assays of cells treated with micromolar EET levels (93, 94). Exogenous treatments with EETs at micromolar concentrations are likely supraphysiologic; however, it is important to note that the efficacy of exogenous EETs may be reduced by EET esterification, EET hydrolysis, and/or the lack of PPAR cofactors in reporter systems (95). PPARγ agonists such as EETs induce expression of the NF-kB inhibitory protein IκBα (93, 96). Since NF-kB is a master pro-inflammatory transcription factor, its inhibition by the EET/PPAR/IκBα axis attenuates expression of pro-inflammatory cytokines and enzymes such as COX-2 (97). Ultimately, many anti-inflammatory effects of EETs may be largely regulated by PPAR-mediated effects, which can suppress endothelial activation, lymphocyte recruitment, atherosclerotic development, cardiac inflammation, and end-stage organ damage (97, 98). PPARγ agonists promote lipogenesis and insulin sensitivity, which have benefits in diabetes (99). EET/PPAR signaling can also regulate vascular tone as PPARγ antagonists disrupt EET-dependent vasodilation in response to laminar flow (93).

EETs bind and activate TRP calcium channels, including TRPA1, TRPC1, TRPC6, and TRPV4, and TRPA1 induces influx of extracellular calcuium (100). While some TRP channels appear most sensitive to 5,6-EET, some are activated by other EpFAs, including 8,9- and 11,12-EET or 19,20-EpDPA (23, 101, 102, 103, 104). One of the originally described functions of EETs was their role as endothelial-derived hyperpolarization factors that induce vascular relaxation (105). While vascular tone can be regulated through GPCR signaling, multiple studies suggest that EETs may hyperpolarize and relax vascular smooth muscle *via* TRP-activated pathways (101, 106). Interestingly, both TRPC and TRPV isoforms appear to act both upstream and downstream of phosphatidyl inositol-3 kinase (PI3K) (100, 107), which is a master regulator of many EET-stimulated effects, including their mitogenic, proliferative, angiogenic, and anti-apoptotic properties (1, 100). The role of TRP activation in the many effects of EET treatment, P450 epoxygenase overexpression, or sEH inhibition have not been extensively investigated.

EETs also bind to and activate Kir6.2-containing K_ATP_ channels, which act as ATP-sensitive redox sensors in cells (108). Sufficient intracellular ATP levels result in ATP binding to the channel, which increases the probability of its closed state. A reduction in ATP levels, which occurs during ischemic events, leads to channel opening (109). While the mechanism remains unclear, K_ATP_ channel opening reduces the catastrophic opening of the mitochondrial permeability transition pore (mPTP) to maintain mitochondrial integrity and membrane potential (110). Both 14,15- and 11,12-EET can displace ATP from K_ATP_ channels and increase their open probabilities. This function of EETs appears critical as K_ATP_ channel inhibitors reverse the protective effect of EETs, P450 overexpression, or sEH disruption on cardiac and cerebral ischemia-reperfusion damage (22, 111, 112, 113).

## Conclusion

Despite intensive and multifaceted efforts to identify high-affinity EET receptors, definitive evidence for such a GPCR remains elusive. While numerous studies suggest that EETs may exert their physiological effects through interactions with known GPCRs, including FFARs (GPR40, GPR120, GPR132), GPR39, thromboxane receptors, and PGE_2_ receptors (EP2, EP4), many of these findings are based on *in vitro* experiments utilizing non-physiological concentrations of the ligand or indirect assays, making extrapolation to *in vivo* relevance challenging. The observed beneficial effects of sEH inhibition in numerous preclinical models of disease further emphasize the need to clarify the downstream signaling cascades of EETs. Future research should prioritize rigorous *in vivo* validation. In particular, genetic knockout models of putative receptors or ion channels should be tested in conjunction with sEH inhibition to definitively establish the contribution of these identified receptors to EpFA-mediated physiology. There is a wide variety of models where the use of sEH null mice or treatment with sEHi dramatically alters physiological outcomes. For example, sEHi improves recovery after cardiac ischemia, reduces blood pressure after angiotensin-2 infusion, accelerates wound healing, and reduces systemic inflammation. If any prostanoid or FFAR receptor knockout mice abolish these effects of sEH inhibition, it would more strongly indicate a role downstream of EETs for these receptors. Such a finding would need confirmation using treatments with individual EpFAs. Furthermore, considering the potential for low-affinity interactions under high local EET concentrations, indirect receptor crosstalk, and the unique membrane localization of EETs, a more nuanced understanding of their signaling mechanisms, which may involve a family of receptors or complex synergistic interactions, will be critical for unlocking the full therapeutic potential of targeting the EET pathway.

## Conflict of interest

The authors declare that they have no conflicts of interest with the contents of this article.
